# Climate variability, poverty and substance use disorders: Exploring maladaptive coping mechanisms in climate-stressed communities of Northwest Nigeria

**DOI:** 10.4102/jphia.v17i1.1665

**Published:** 2026-07-20

**Authors:** Abdullahi Y. Abdulwahab, Summayyah B. Muhammad, Audu I. Aveka, Abdulkadir Surajudeen, Sani Khalid, Muhammad Aliyu, Abusufyan Attahiru, Fahad S. Abubakar, Bashir A. Yakasai

**Affiliations:** 1 Department of Psychiatry, Faculty of Clinical Sciences, Ahmadu Bello University, Zaria, Nigeria; 2 Department of Psychiatry, Faculty of Clinical Sciences, Ahmadu Bello University Teaching Hospital, Zaria, Nigeria; 3 Department of Clinical Services, Faculty of Clinical Sciences, Federal Neuropsychiatric Hospital, Kaduna, Nigeria; 4 Department of Psychiatry, Faculty of Clinical Sciences, Usmanu Danfodio University Teaching Hospital, Sokoto, Nigeria; 5 Department of Community Medicine, Faculty of Clinical Sciences, Ahmadu Bello University Teaching Hospital, Zaria, Nigeria

**Keywords:** climate change, coping mechanisms, mental health, substance use, poverty, Nigeria, syndemic, climate variability

## Abstract

**Background:**

Northwest Nigeria is highly vulnerable to climate variability and heavily dependent on rain-fed agriculture, which exacerbates existing socioeconomic vulnerabilities.

**Aim:**

This study aimed to explore the link among climate-induced stress, poverty, and mental health outcomes, particularly substance use as a maladaptive coping mechanism, in this region.

**Setting:**

The study was conducted in Kano, Kaduna, and Katsina in Northwest Nigeria.

**Methods:**

A cross-sectional study was conducted among 20 000 adults aged ≥ 18 years from 40 randomly selected communities. Data were collected using validated instruments: The Alcohol, Smoking and Substance Involvement Screening Test (ASSIST), the Kessler Psychological Distress Scale (K10), the Coping Strategies Inventory-Short Form (CSI-SF), and a Multidimensional Poverty Index Questionnaire. Robust statistical analyses, including multivariable logistic regression with cluster-robust standard errors, were employed.

**Results:**

The prevalence of high-risk substance use (ASSIST ≥ 27) was 18.5% (*n* = 3700). Cannabis was the most commonly misused substance (12.4%, *n* = 2480), followed by prescription opioids (9.1%, *n* = 1820) and alcohol (7.8%, *n* = 1560). High psychological distress (K10 ≥ 20) was identified in 45.2% (*n* = 9040). Over 85% (*n* = 17 000) were multidimensionally poor. Maladaptive coping strategies were strongly correlated with both poverty (odds ratio [OR]: 3.12, 95% confidence interval [CI]: 2.85–3.42) and high psychological distress (OR: 4.85, 95% CI: 4.48–5.25).

**Conclusion:**

This study reveals a concerning syndemic of climate-related stress, profound poverty, poor mental health, and substance use in Northwest Nigeria. Associations suggest substance use may function as a maladaptive coping mechanism in the context of climate-induced economic hardship, though causal inference is limited by the cross-sectional design.

**Contribution:**

Findings underscore the critical need for integrated public health interventions that address mental health and substance use disorders within the context of climate adaptation and poverty reduction strategies.

## Introduction

The accelerating pace of climate change presents one of the most significant threats to global health in the 21st century, with impacts disproportionately affecting the world’s most vulnerable populations.^1^ Northwest Nigeria, situated within the arid and semi-arid Sahelian region, is a quintessential example of a climate-stressed hotspot. The region is characterised by increasing temperature variability, erratic rainfall patterns, prolonged droughts and desertification, which collectively undermine the agrarian and pastoral livelihoods upon which a majority of the population depends.^2,3^ These environmental shocks act as potent drivers of food insecurity, economic instability and forced migration, creating a cascade of socioeconomic vulnerabilities.^4^

Poverty, both a cause and consequence of climate vulnerability, is endemic in Northwest Nigeria. The region reports some of the highest multidimensional poverty indices in the country, with limited access to education, healthcare and secure employment.^5^ This socioeconomic precarity creates a fertile ground for poor mental health outcomes. The psychological toll of climate variability – encompassing loss of livelihood, displacement and uncertainty about the future – has been associated with anxiety, depression and profound stress.^6,7^ Globally, a growing body of literature has established a strong association between exposure to extreme weather events, gradual environmental degradation and adverse mental health consequences, often termed ‘eco-anxiety’ or ‘solastalgia’ (the distress caused by environmental change).^8,9^

Confronted with such chronic stressors and lacking access to formal mental health support or adaptive economic resources, individuals may turn to maladaptive coping mechanisms. Substance use, particularly the misuse of alcohol, cannabis and prescription opioids, has been identified as a common, though detrimental, strategy to self-medicate psychological distress and escape the realities of hardship.^10,11^ This may create a vicious cycle: substance use can exacerbate poor mental health, impair economic productivity and deepen poverty, further reducing resilience to future climate shocks.^12^

While studies have explored fragments of this complex nexus – climate and migration, poverty and mental health, or mental health and substance use in other contexts^13,14^ – there is a critical paucity of research that integrates these strands within the unique socio-ecological context of Northwest Nigeria. This study, therefore, seeks to address this gap. We hypothesise that climate variability acts as a foundational stressor that, mediated through extreme poverty and resulting psychological distress, is associated with the adoption of maladaptive coping strategies, notably substance use disorders. This study aims to quantify the prevalence of these issues and elucidate their interrelationships within a large, representative sample from Northwest Nigeria, providing crucial evidence for targeted public health and policy interventions.

## Research methods and design

### Study design and setting

A community-based cross-sectional study was conducted between January 2024 and April 2024 in three states (Kano, Kaduna and Katsina) in Northwest Nigeria. This region was selected because of its high exposure to climate variability and its high ranking on national poverty indices.

### Participants

Participants were adult (aged 18 years or older) residents of Kano, Kaduna and Katsina States, who gave informed written consent to participate in the study.

### Inclusion criteria

The inclusion criteria included those who were aged 18 years or older, permanent residents of the selected community (≥ 6 months) and provided informed consent.

### Exclusion criteria

The exclusion criteria included those who were severely ill or cognitively impaired to the extent that they could not participate in the interview, and visitors temporarily residing in the community.

### Sample size determination

The sample size was calculated using the formula for estimating a single proportion,^15^ with a conservative prevalence (*p*) of substance use of 50% (to yield the maximum sample size), a margin of error (*d*) of 0.007, a design effect of 2 and a 95% confidence level (*Z* = 1.96). This calculation yielded a minimum sample of 15 747. This was rounded up to 20 000 to account for potential non-response and to allow for robust subgroup analyses.

### Sampling method

A multi-stage stratified random sampling technique was employed. First of all, 40 communities (clusters) were randomly selected from a comprehensive list of communities across the three states, stratified by urban and rural location. Within each selected community, 500 households were randomly selected using a modified random walk procedure.^16^ One eligible adult (aged 18–65 years) was randomly selected from each household using the Kish grid method^17^ to minimise selection bias.

### Conceptual framework

In this study, we conceptualise a pathway whereby climate variability (contextual stressor) exacerbates multidimensional poverty, which in turn increases psychological distress. This distress, compounded by a lack of access to adaptive coping resources, is associated with the use of maladaptive coping strategies, most notably high-risk substance use. Coping strategy type (adaptive vs. maladaptive) is described as a population characteristic; however, in the regression model, poverty and psychological distress are treated as independent variables predicting high-risk substance use. Maladaptive coping is not entered as a separate predictor to avoid conceptual overlap with the outcome. This framework is descriptive and associative, not causal, given the cross-sectional design.

### Study instruments

- **Socio-demographic and poverty questionnaire:** It collected data on age, gender, education, occupation and income. Poverty was assessed using a multidimensional index adapted from the Nigerian National Bureau of Statistics,^5^ encompassing indicators of health, education and living standards.
- **Alcohol, Smoking and Substance Involvement Screening Test (ASSIST):** It is used to assess the use of psychoactive substances (tobacco, alcohol, cannabis, cocaine, stimulants, sedatives, hallucinogens, inhalants and prescription opioids) and related risks. A score of ≥ 27 for any substance indicates a high risk of dependence and need for treatment.^18^ For participants using multiple substances, the highest substance-specific score was used to determine high-risk status. Prevalence by substance type reports any use of that substance at high-risk level; therefore, categories are not mutually exclusive. The ASSIST has been validated for use in Nigeria, demonstrating good reliability (Cronbach’s alpha > 0.80).^19^
- **Kessler Psychological Distress Scale (K10):** It is a 10-item instrument measuring levels of anxiety and depressive symptoms in the past 30 days. Scores range from 10 to 50; a score of ≥ 20 indicates a high likelihood of a severe mental disorder.^20^ The K10 has been widely used and validated in Nigeria, showing high internal consistency (alpha = 0.89).^21^
- **Coping Strategies Inventory – Short Form (CSI-SF):** This 16-item scale measures engagement and disengagement coping strategies, further categorised into adaptive and maladaptive coping. Higher scores on the maladaptive subscale indicate a greater reliance on avoidance and emotional discharge.^22^ Its use in Nigerian populations has shown good psychometric properties.^23^

### Study procedure

Trained research assistants, fluent in both English and Hausa, conducted face-to-face interviews using structured questionnaires translated into Hausa and back-translated (using the WHO iterative back translation technique) to ensure conceptual accuracy.

### Statistical analysis

Data were analysed using Stata version 18.0. Descriptive statistics (frequencies, percentages, means and standard deviations) were used to summarise socio-demographic characteristics and key variables. Chi-square tests were used to examine associations between categorical variables. To account for potential correlation within communities, we used cluster-robust standard errors with community as the primary sampling unit (Stata vce [cluster community]). Covariates (poverty, psychological distress, age, gender and unemployment) were selected *a priori* based on the existing literature and entered simultaneously into the multivariable model; no stepwise selection methods were used. Variance inflation factors (VIFs) were computed to assess multicollinearity; all VIFs were below 2.5, indicating no significant multicollinearity. Univariable and multivariable logistic regression analyses were performed to identify factors associated with high-risk substance use (ASSIST score ≥ 27), with results presented as odds ratios (OR) and 95% confidence intervals (CI). A *p*-value of < 0.05 was considered statistically significant.

### Ethical considerations

Ethical clearance to conduct this study was obtained from the Ahmadu Bello University Teaching Hospital research ethics committee (No. ABUTH/HREC/C37/2024). Informed written consent was obtained from all participants prior to their inclusion in the study. Confidentiality and anonymity of participants were maintained throughout the research process. Participants were duly informed they could withdraw from the study at any time without any consequences. Participants who screened positive for high-risk substance use (ASSIST ≥ 27) or severe psychological distress (K10 ≥ 30) were provided with a list of local mental health and substance use support services, including the nearest primary health centre with a trained community health extension worker, and referral contacts for the Federal Neuro-Psychiatric Hospital, Kaduna. Transport reimbursement for referral follow-up was offered where feasible.

## Results

The findings from this large-scale study of 20 000 individuals across Northwest Nigeria reveal a deeply concerning and interconnected web of socioeconomic disadvantage, psychological distress and maladaptive health behaviours.

As detailed in Table 1, the socio-demographic profile of the sample is characteristic of a vulnerable, agrarian society. A significant proportion of respondents were young adults, with those aged 18–35 years constituting 57.5% (*n* = 11 500) of the sample. This is a critical demographic, as youth are both the most valuable asset for a region’s future and, as the results will show, highly vulnerable to the documented risks. The educational attainment was low, with 42.5% (*n* = 8500) having received no formal education. Almost half of the sample (45.0%, *n* = 9000) were engaged in farming or herding, occupations directly threatened by climate variability in Northwest Nigeria.

**TABLE 1:** Socio-demographic characteristics of participants (*N* = 20 000).

Characteristic	Category	Frequency (*n*)	Percentage (%)
Age group (years)	18–25	5200	26.0
	26–35	6300	31.5
	36–45	4500	22.5
	46–55	2600	13.0
	56–65	1400	7.0
Gender	Male	9580	47.9
	Female	10 420	52.1
Education	No formal education	8500	42.5
	Primary	6200	31.0
	Secondary	3800	19.0
	Tertiary	1500	7.5
Occupation	Farming/herding	9000	45.0
	Unemployed	4500	22.5
	Informal trade	4800	24.0
	Skilled employment	1700	8.5

Table 2 presents the core findings. The extent of multidimensional poverty is profound, affecting 85.0% (*n* = 17 000) of participants. This figure exceeds the national average and underscores the extreme economic precarity in this region. The Kessler-10 scale identified that 45.2% (*n* = 9040) of respondents experienced high levels of psychological distress, a prevalence that indicates a population-wide mental health burden. The data show that 67.5% (*n* = 13 500) of respondents primarily utilised maladaptive coping strategies, as measured by the CSI-SF. The ASSIST tool identified that 18.5% (*n* = 3700) of the entire sample were engaged in high-risk substance use. Cannabis was the most prevalent substance of misuse (12.4%, *n* = 2480). The high use of prescription opioids (9.1%, *n* = 1820) is particularly concerning because of the associated risks of dependence and overdose. Alcohol use (7.8%, *n* = 1560) and solvent use (4.8%, *n* = 950) further compound this picture.

**TABLE 2:** Prevalence of key study variables.

Variable	Category	Frequency (*n*)	Percentage (%)
Multidimensional poverty	Poor	17 000	85.0
	Not poor	3000	15.0
Psychological distress (K10)	Low (10–19)	10 960	54.8
	High (≥ 20)	9040	45.2
Substance use (ASSIST high risk)	Any substance	3700	18.5
	**By type:**		
	Cannabis	2480	12.4
	Prescription opioids	1820	9.1
	Alcohol	1560	7.8
	Solvents or inhalants	950	4.8
	**Coping strategy (CSI-SF)**		
	Primarily adaptive	6500	32.5
	Primarily maladaptive	13 500	67.5

The multivariable logistic regression analysis presented in Table 3 synthesises these relationships. After adjusting for other covariates, individuals living in multidimensional poverty had over three times the odds (adjusted odds ratio [aOR]: 3.12, 95% CI: 2.85–3.42) of being a high-risk substance user compared to their non-poor counterparts. Individuals with high K10 scores had nearly five times the odds (aOR: 4.85, 95% CI: 4.48–5.25) of high-risk substance use. Being male was associated with 2.5 times the odds (aOR: 2.50, 95% CI: 2.31–2.70) of substance misuse. Younger adults (aged 18–35 years) were significantly more likely to engage in high-risk use compared to the oldest age group (56–65 years). Unemployment also emerged as a significant independent risk factor (aOR: 1.75, 95% CI: 1.60–1.92).

**TABLE 3:** Factors associated with high-risk substance use (multivariable logistic regression).

Factor	Adjusted odds ratio (aOR)	95% confidence interval	*p*-value
Poverty (Ref: Not poor)	3.12	2.85–3.42	< 0.001
High psychological distress (Ref: Low)	4.85	4.48–5.25	< 0.001
Male gender (Ref: Female)	2.50	2.31–2.70	< 0.001
**Age group (years) (Ref: 56–65)**			
18–25	1.90	1.62–2.23	< 0.001
26–35	2.15	1.84–2.51	< 0.001
Unemployed (Ref: Employed)	1.75	1.60–1.92	< 0.001

Ref, reference.

## Discussion

This large-scale study provides evidence of a concerning syndemic in Northwest Nigeria, where climate-induced socioeconomic stress, profound poverty, high rates of mental illness and substance use disorders co-occur and may interact synergistically. These conditions do not merely co-occur; they may interact and mutually reinforce one another. Poverty increases vulnerability to psychological distress, which in turn is associated with elevated risk of substance use as a maladaptive coping strategy. Substance use may then deepen poverty and impair resilience to further climate shocks, potentially creating a self-perpetuating cycle. However, causal inference is limited by the cross-sectional design.

The finding that 85% of our sample was multidimensionally poor is consistent with national data^5^ but underscores the extreme deprivation in this region. This figure aligns with subnational estimates from the 2022 Nigerian Multidimensional Poverty Index (MPI) Report, which range from 65% to 90% across Northwest zones, confirming that our sample is regionally representative rather than anomalously high. This poverty is embedded in a context of environmental degradation, which limits opportunity and erodes resilience. Our results indicate that poverty is associated with psychological distress, with 45.2% of respondents scoring highly on the K10. This prevalence is higher than the estimated 20% – 30% prevalence of common mental disorders reported in general primary care settings in Nigeria,^24^ suggesting that climate-stressed communities may represent a high-risk subgroup. Nonetheless, self-reported distress may be subject to reporting bias, and the lack of diagnostic clinical interviews means these figures represent symptom burden rather than clinical caseness.

The prevalence of high-risk substance use (18.5%), particularly cannabis and prescription opioids, is concerning. This finding aligns with emerging reports from Northern Nigeria indicating a rising trend in substance misuse, especially among youth.^25,26^ The use of prescription opioids like tramadol, often acquired without a prescription, is of particular public health concern because of its association with dependence and overdose.^27^ Our regression analysis confirms that poverty and psychological distress are potent, independent predictors of substance use. This suggests that substance use may be employed as a maladaptive coping mechanism to alleviate distress associated with economic hardship – a phenomenon observed in other stressed populations globally.^11,28^

The predominance of maladaptive coping strategies (67.5%) further reinforces this interpretation. When faced with substantial stressors and lacking access to mental health services or alternative livelihoods, communities may resort to avoidance and emotional discharge, of which substance use is a prime example. This may create a cycle whereby substance use further impairs functioning and deepens poverty.

Our findings resonate with studies from other parts of Africa. In Kenya, researchers linked drought conditions to increased stress and alcohol use among pastoralists.^29^ In South Africa, poverty and unemployment have been consistently linked to substance use and mental health problems.^30^ Globally, a meta-analysis confirmed that exposure to climate-related disasters and environmental change is associated with increased rates of depression, anxiety and substance use.^9^ This study contributes to this global literature by quantifying these relationships in the specific and highly vulnerable context of the Sahel.

The following findings are directly supported by the data presented: (1) prevalence estimates of multidimensional poverty (85%), psychological distress (45.2%), maladaptive coping (67.5%) and high-risk substance use (18.5%); (2) aOR showing independent associations between poverty, distress, male gender, younger age, unemployment and high-risk substance use. The hypothesised pathway involving climate variability as an underlying driver is contextually plausible but not directly tested.

Based on these associational findings, the following policy implications are offered: (1) integrate mental health and substance use prevention into climate adaptation and food security programmes; (2) scale up social protection and livelihood diversification; (3) train community health workers to identify and refer individuals with mental health and substance use issues; (4) commission longitudinal studies to establish temporal causality and explore culturally relevant adaptive coping mechanisms.

## Conclusion

This study reveals a critical public health challenge in Northwest Nigeria. Because poverty, psychological distress and substance use share underlying determinants – economic precarity, environmental instability and a lack of mental health infrastructure – siloed interventions are unlikely to succeed. Addressing substance use in isolation will be ineffective. An integrated, syndemic approach is required.

### Recommendations

We recommend that authorities should mainstream mental health and substance use prevention into climate adaptation and food security programmes; scale up social protection schemes and livelihood diversification programmes to build economic resilience; train community health workers to identify, support and refer individuals with mental health and substance use issues, overcoming the shortage of specialists; and commission longitudinal studies to better establish causality and explore culturally relevant adaptive coping mechanisms.

### Limitations

The cross-sectional design precludes definitive causal inferences. While we hypothesise climate variability as a root cause, its effect was measured indirectly through its impacts on livelihood and poverty. Climate variability was not directly measured using meteorological or remote sensing indices; rather, its role is inferred from regional ecological context and documented impacts on agrarian livelihoods. Future studies should integrate objective climate exposure data. Self-reported data may be subject to social desirability bias, potentially leading to underreporting of substance use and mental health symptoms. Selection of households with permanent residents (≥ 6 months) may exclude highly mobile individuals, potentially underestimating substance use among migrant or transient populations.
